# Multiplexing detection of IgG against *Plasmodium falciparum* pregnancy-specific antigens

**DOI:** 10.1371/journal.pone.0181150

**Published:** 2017-07-17

**Authors:** Ana Maria Fonseca, Llorenç Quinto, Alfons Jiménez, Raquel González, Azucena Bardají, Sonia Maculuve, Carlota Dobaño, Maria Rupérez, Anifa Vala, John J. Aponte, Esperanza Sevene, Eusebio Macete, Clara Menéndez, Alfredo Mayor

**Affiliations:** 1 ISGlobal, Barcelona Ctr. Int. Health Res. (CRESIB), Hospital Clínic - Universitat de Barcelona, Barcelona, Spain; 2 Graduate Program in Areas of Basic and Applied Biology (GABBA), Universidade do Porto, Porto, Portugal; 3 Spanish Consortium for Research in Epidemiology and Public Health (CIBERESP), Madrid, Spain; 4 Centro de Investigação em Saúde da Manhiça (CISM), Maputo, Mozambique; 5 Eduardo Mondlane University, Maputo, Mozambique; Ehime Daigaku, JAPAN

## Abstract

**Background:**

Pregnant women exposed to *Plasmodium falciparum* generate antibodies against VAR2CSA, the parasite protein that mediates adhesion of infected erythrocytes to the placenta. There is a need of high-throughput tools to determine the fine specificity of these antibodies that can be used to identify immune correlates of protection and exposure. Here we aimed at developing a multiplex-immunoassay to detect antibodies against VAR2CSA antigens.

**Methods and findings:**

We constructed two multiplex-bead arrays, one composed of 3 VAR2CSA recombinant-domains (DBL3X, DBL5Ɛ and DBL6Ɛ) and another composed of 46 new peptides covering VAR2CSA conserved and semi-conserved regions. IgG reactivity was similar in multiplexed and singleplexed determinations (Pearson correlation, protein array: R^2^ = 0.99 and peptide array: R^2^ = 0.87). IgG recognition of 25 out of 46 peptides and all recombinant-domains was higher in pregnant Mozambican women (n = 106) than in Mozambican men (n = 102) and Spanish individuals (n = 101; p<0.05). Agreement of IgG levels detected in cryopreserved plasma and in elutions from dried blood spots was good after exclusion of inappropriate filter papers. Under heterogeneous levels of exposure to malaria, similar seropositivity cutoffs were obtained using finite mixture models applied to antibodies measured on pregnant Mozambican women and average of antibodies measured on pregnant Spanish women never exposed to malaria. The application of the multiplex-bead array developed here, allowed the assessment of higher IgG levels and seroprevalences against VAR2CSA-derived antigens in women pregnant during 2003–2005 than during 2010–2012, in accordance with the levels of malaria transmission reported for these years in Mozambique.

**Conclusions:**

The multiplex bead-based immunoassay to detect antibodies against selected 25 VAR2CSA new-peptides and recombinant-domains was successfully implemented. Analysis of field samples showed that responses were specific among pregnant women and dependent on the level of exposure to malaria. This platform provides a high-throughput approach to investigating correlates of protection and identifying serological markers of exposure for malaria in pregnancy.

## Introduction

Serological studies provide useful data for the identification of immune correlates of protection against infectious pathogens [1] and for the epidemiological surveillance of the infection [2]. The measurement of antibodies in plasma can guide the selection of malaria vaccine candidates through the identification of antibodies that last long in the blood and correlate with protection. As antibodies can circulate for weeks to months after active malaria infections, they can also be used to determine the history of exposure over time. Such serological biomarkers of malaria exposure can allow a more sensitive quantification of low malaria transmission when detecting the parasite becomes impractical due to the need for large sample sizes [3].

Malaria in pregnancy is characterized by the sequestration of infected erythrocytes in the placenta through the adhesion to chondroitin sulfate-A on syncytiotrophoblasts [4]. Adhesion is mediated by VAR2CSA [5], a member of the *P*. *falciparum* erythrocyte membrane protein 1 (PfEMP1) family encoded by the *var* multigene family [6]. VAR2CSA is a large protein of 350 kDa composed of an intracellular and an extracellular region organized in 6 Duffy binding-like domains (3DBLX and 3DBLƐ) [4]. Among the members of the *var* multigene family, *var2csa* presents the lowest variability between parasite strains, with nucleotide pairwise identity from 54% to 94% [7,8] and aminoacid identity average of 78% (range 75–83%) [9].

Anti-VAR2CSA antibodies are acquired after exposure to *P*. *falciparum* infection during pregnancy [10], increase with successive pregnancies (parity-dependent) [11] and cross-react between geographically diverse placental isolates suggesting conserved epitopes [12], overlap of polymorphisms [9,13] or polymorphic conformational epitopes [14]. High levels of antibodies against VAR2CSA have been associated with reduced risk of placental infection [15–17], but also with low birth weight [6,16,18–20], and maternal anemia [19], supporting their dual role in protection and as marker of exposure. Anti-VAR2CSA antibodies have also been shown to mirror changes in the prevalence of infection in pregnant women from a rural area in Mozambique [21], suggesting the potential of these antibodies to provide information about changing trends in malaria transmission intensity [22,23].

Measuring antibodies against native VAR2CSA protein present in the surface of infected erythrocytes by flow cytometry [12] and recombinant proteins by ELISA (enzyme linked immunosorbent assay) [16,20,24] is time-consuming and does not allow dissection of the fine specificity of interactions involved in immune protection. As a result of the growing demand for rapid, precise and cost-effective techniques for the detection of antibodies, multiplex-bead array assays have been developed to provide measures using large numbers of antigens [25,26]. However, very few studies have used multiplex assays to measure antibodies against malaria antigens specifically expressed during pregnancy [17,27–30].

We aimed at developing a multiplex-bead array assay to detect antibodies against a panel of new peptides covering VAR2CSA and recombinant domains that could be used for the identification of immune correlates of protection against placental malaria and its adverse consequences, as well as to identify serological markers for surveillance of malaria transmission. To achieve this, antigens were coupled to microspheres, and the effect of multiplexing as well as the assay performance were assessed using plasma samples collected from a population in South Mozambique and Spanish individuals never exposed to malaria. VAR2CSA peptides were selected based on the pregnancy-specific recognition by pregnant women naturally exposed to malaria in Mozambique, compared to non-pregnant Mozambican adults. We tested this serological platform on plasma samples but also on blood collected on filter paper as a convenient alternative for serological surveys in remote locations [31]. We also compared the use of plasmas from pregnant women never exposed to malaria [27] and finite mixture models (FMM) [32] to provide seropositivity thresholds. Finally, we applied the multiplex-bead array to measure IgG responses in pregnant Mozambican women from two time periods (2003–2005 and 2010–2012) with different intensities of malaria transmission [21].

## Methodology

### Ethics statement

The Mozambican National Health and Bioethics Committee and the Hospital Clinic of Barcelona Ethics Committee approved the study and the use of non-identifiable plasma and serum samples for the immunological studies described [33–36]. Written informed consent was obtained from all the adult participants and from parents or guardians of healthy children participating in the RTS,S vaccine study.

### Antigens

Recombinant proteins used were VAR2CSA Duffy binding-like domains (DBL3X, DBL5Ɛ and DBL6Ɛ, from 3D7 strain) [20,37], apical membrane antigen 1 (AMA1, from 3D7 strain) [38], merozoite surface protein-1, 19-kDa, (MSP1_19_, from 3D7 strain) [39], all produced at ICGEB (New Delhi, India); circumsporozoite protein (rCSP) from *P*. *falciparum* purchased from Sanaria, Rockville; and *Clostridium tetani*, tetanus toxin purchased from Santa Cruz Biotechnology (Dallas, Texas). Forty-six synthetic peptides covering conserved and semi-conserved regions from VAR2CSA were designed after alignment of 18 full-length sequences from different geographic origins (Asia, Africa, Central and South America; Table A in S1 File and design strategy detailed in Text A in S1 File). A circumsporozoite peptide (pCSP) of 64 aminoacids (NVDP[NANP]_15_) was also included (adapted from [40]). Peptides were synthesized by GL Biochem (Xangai, China) and median purity was estimated as 79% (range: 71–91%) by HPLC (high performance liquid chromatography) and mass spectrometry.

### Development of multiplex arrays to measure IgG

Two multiplex suspension array panels were constructed to quantify IgG responses against *P*. *falciparum* recombinant proteins and synthetic peptides, using the xMAP ^™^ technology and the Luminex^®^ 100/200^™^ System (Luminex^®^ Corp., Austin, Texas). MagPlex^®^ microspheres (magnetic carboxylated polystyrene microparticles, 5.6 μm) with different spectral signatures were selected for each protein (DBL3X, DBL5Ɛ, DBL6Ɛ, AMA1, MSP1_19_ and rCSP), peptide (46 VAR2CSA peptides and pCSP), tetanus toxin and bovine serum albumin (BSA). Antigens were covalently coupled to beads following a modification of the Luminex^®^ Corporation protocol (Text B in S1 File). Antigen amounts in the coupling reaction for one million beads were determined after a titration experiment, and were as follows: 2 μg of rCSP and tetanus toxin, 4 μg of DBL3X, DBL5Ɛ, DBL6Ɛ, AMA1, 8 μg of MSP1_19_, 170 μg of each peptide and for BSA a 1% solution in PBS. To determine if coupling was effective, beads were tested with two hyperimmune plasma pools (HIP). One pool was composed of 23 plasmas from pregnant Mozambican women hyperimmune against VAR2CSA (HIP-VAR2CSA) and the other was composed of 6 RTS,S/AS02-vaccinated children [41] hyperimmune against the circumsporozoite (CSP) protein (Luminex^®^ assay described in Text C in S1 File). Protein and peptide multiplex arrays were prepared by pooling together equal volumes of coated beads. All samples were analyzed in duplicate at dilution 1:400 for the protein array and 1:100 for the peptide array. A positive control (HIP-VAR2CSA) was included in each assay plate, in addition to blanks (wells without plasma sample) to assess background levels. A minimum of 50 microspheres were read per spectral signature and results were exported as crude median fluorescent intensity (MFI). Duplicates were averaged and background MFIs were subtracted. Results were normalized (nMFI) to account for plate-to-plate variation by dividing the background subtracted MFI of each sample by the value of the positive pool in the same plate and multiplying with the median of positive pools in all plates.

### Study population

For the development of both multiplex arrays, we measured IgGs in 550 pregnant women (550 plasma samples [106/550 immune against VAR2CSA as assessed by flow cytometry against native VAR2CSA expressed on CS2 parasite line] and 240 dried blood spots [DBS]) residing in Manhiça district (Southern Mozambique) who participated in two clinical trials of intermittent preventive treatment conducted between 2003–2005 [35] and 2010–2012 [33,34] (Table 1).

**Table 1:** Characteristics of pregnant Mozambican women included in the study.

Variable	VAR2SSA-immune^¶^	2003–2005	2010–2012	p^§^
	N = 106	N = 204	N = 240	
***Parity*, *n (%)***				
Primigravidae	10 (9)	55 (27)	70 (29)	0.607
Multigravidae	96 (91)	149 (73)	170 (71)	
***Age*, *n (%)***				
<20yr	22 (21)	64 (31)	69 (29)	0.195
20-24yr	31 (29)	59 (29)	56 (23)	
≥25yr	53 (50)	81 (40)	115 (48)	
***HIV status*, *n (%)***				
Negative	81 (76)	144 (71)	197 (82)	0.004
Positive	25 (24)	60 (29)	43 (18)	
*Malaria status*, n(%)*				
Negative	24(36)^#^	125 (61)	228 (95)	<0.001
Positive	43 (64)	79 (39)	12 (5)	

^¶^ Immune pregnant women from Mozambique against the VAR2CSA native protein measured by cytometry using the CS2 laboratory strain, collected between 2003–2005

*Positive if peripheral and/or placental blood were positive for *P*. *falciparum* by qPCR targeting the 18s rRNA

^#^ qPCR (periphery and placenta) performed in 67 (63%) of 106 samples CS2 IgG positive

^§^ Comparison between pregnant women from 2003–2005 with 2010–2012 by Fisher exact test

Manhiça is a region in southern Mozambique under continuous demographic surveillance by the Centro de Investigação em Saúde de Manhiça (CISM) [42]. Pregnancy-associated malaria in this region used to be intense (2003–2005) and has decreased in the past few years (2010–2012) [21]. Parasite detection by qPCR and anti-CS2 IgG levels were measured in a previous study [21]. Plasma samples from 102 men (50% malaria positive by microscopy) recruited at the Manhiça District Hospital during 2006 were included [43,44]. In addition, 49 plasma samples were collected during 2010 from pregnant women recruited at delivery at the Hospital Clinic in Barcelona and 52 were collected during 2008 from healthy adult men in Barcelona. Finally, we also included serum samples from 6 RTS,S/AS02-vaccinated children obtained during scheduled study visits as specified per protocol to measure antibodies against CSP antigen.

Plasma and serum samples were cryopreserved at -80°C. DBS were prepared and stored avoiding humidity at -20°C.

### Reconstitution of dried blood spots

DBS collected from 240 pregnant Mozambican women, as well as freshly prepared DBS using plasma from positive and negative controls resuspended in full blood, were tested for antibody elution. Briefly, four spots of approximately 3 mm in diameter were cut from the filter papers using a punch (McGill^®^ round punch, 3 mm) and transferred to individual wells of a 96-well polystyrene U-bottom plate. Antibodies were eluted with 200 μl Luminex^®^ assay buffer (1% BSA, 0.05% sodium azide in filtrated PBS [Phosphate-buffered saline]) at room temperature overnight with gentle mixing which, assuming a hematocrit of 50% gives a concentration of eluted blood proteins equivalent to a 1:50 plasma dilution (adapted from [32,45]). To assess the quality of the elution, hemoglobin levels in the eluted DBS were measured by spectrophotometry (wavelengths 415, 380 and 450) and calculated using the Harboe method with the Allen correction (Hb[mg/l] = 167.2 x A415–83.6 x A380–83.6 x A450) x dilution factor). Three criteria to discard DBS improperly eluted were established. First, reddish-brown spots against a pale background were discarded after visual examination of spots reconstitution [32]. Second, DBS were also discarded if hemoglobin levels measured in the elutions were below the upper quartile of hemoglobin value among samples considered with inappropriate visual aspect. Finally, samples were also discarded if anti-tetanus antibodies measured in the elutions were below the lowest quartile obtained from anti-tetanus IgG among samples with appropriate visual aspect and hemoglobin levels. To test if DBS can be considered an alternative to plasma, antibodies eluted from filter paper spots and correspondent antibodies from cryopreserved plasma were measured against the protein array and a subset of the peptide array (15 VAR2CSA-based peptides + pCSP).

### Definition of seropositives

Pregnant women were classified as seropositive against the recombinant domains and peptides by 2 methods: 1) nMFI above the mean plus 3 standard deviations (SD) from 49 pregnant Spanish women never exposed to malaria; 2) nMFI above the mean plus 3 SD from a seronegative population of pregnant Mozambican women delivering between 2003–2012 identified by a FMM that uses maximum likelihood estimation to fit a two-component model of seronegatives and seropositives [32]. To assess the performance of the model to fit the two-components in samples from pregnant Mozambican women from different malaria transmission intensities, the FMM was calculated from nMFIs against the protein array and a subset of the peptide array (15 VAR2CSA-based peptides + pCSP) obtained among plasma samples collected during 2003 to 2005 as a period of intense malaria transmission (high, n = 204), 2010 to 2012 as a period of low malaria transmission (low, n = 240) and from a group of samples from both periods (high & low, n = 444) (samples detailed in Table 1) [21].

### Statistical analysis

Intra-assay variation was calculated as the mean coefficient of variation (CV) from replicates of 7 plasma samples analyzed in each of the 37 plates. The inter-assay variation was calculated as the CV of the median MFI from all antigens included in each multiplex array measured in the positive pool (HIP-VAR2CSA) repeated in the 37 consecutive plates, before normalization [46,47]. Data was fitted to a normal distribution by logarithmic transformation of nMFIs. Pearson`s rank coefficient was used to assess the correlation between nMFI values for each antigen in singleplex versus multiplex. nMFIs were compared between study groups through linear regression analysis. Agreement of nMFIs measured in plasma and in correspondent blood eluted from DBS was assessed by Bland-Altman plots. Results were expressed as bias (mean of the differences) and 95% confidence level of agreement wide (CLw) and interpreted as suggested by Giavarina et all [48]. Agreement of seroprevalence defined by cutoffs calculated using nMFI values from pregnant women never exposed to malaria or FMM were evaluated by Kappa statistic and interpreted as suggested by Landis and Koch [49]. Proportions were compared by Fisher’s exact test. Linear and logistic regression models adjusted by age, parity and HIV were estimated to compare nMFIs and seroprevalences obtained from pregnant Mozambican women recruited during 2003–2005 and 2010–2012. Statistical analyses were performed with Stata/SE software (version 12.0; StataCorp) and Graphpad Prism (version 6, Graphpad, Inc). P-values of less than 0.05 were considered to indicate statistical significance.

## Results

### Characteristics of VAR2CSA peptides

The alignment of 18 VAR2CSA aminoacid sequences yielded 34% positions that were polymorphic with positional Shannon entropy values above 0.43 (second tercile) (Figure A in S1 File). On average, DBL3X and DBL5Ɛ domains showed lower entropy (mean entropy [SD] of 0.20 [0.28] for DBL3X and 0.21 [0.31] for DBL5Ɛ) than DBL6Ɛ (0.52 [0.50]). Forty-six synthetic peptides with lengths between 35 to 65 amino acids from conserved and semi-conserved regions of VAR2CSA were designed for the multiplex-bead array (Figure A in S1 File and Table B in S1 File). Five peptides were from the DBL1X domain, 4 from DBL2X, 6 from DBL3X, 6 from DBL4Ɛ, 5 from DBL5Ɛ, 4 from DBL6Ɛ and 16 from N-terminal segment (NTS) and inter-domain regions (ID). Peptides corresponding to the C-terminal region of the protein (ID5-DBLƐ-ID6) showed on average higher entropy values than peptides from the other regions (mean entropy [SD] from C-terminal region = 0.37 [0.42] and mean entropy [SD] from other regions = 0.15 [0.26]; p<0.001).

### Coupled beads are recognized by IgG from hyperimmune samples

**Fig 1:**
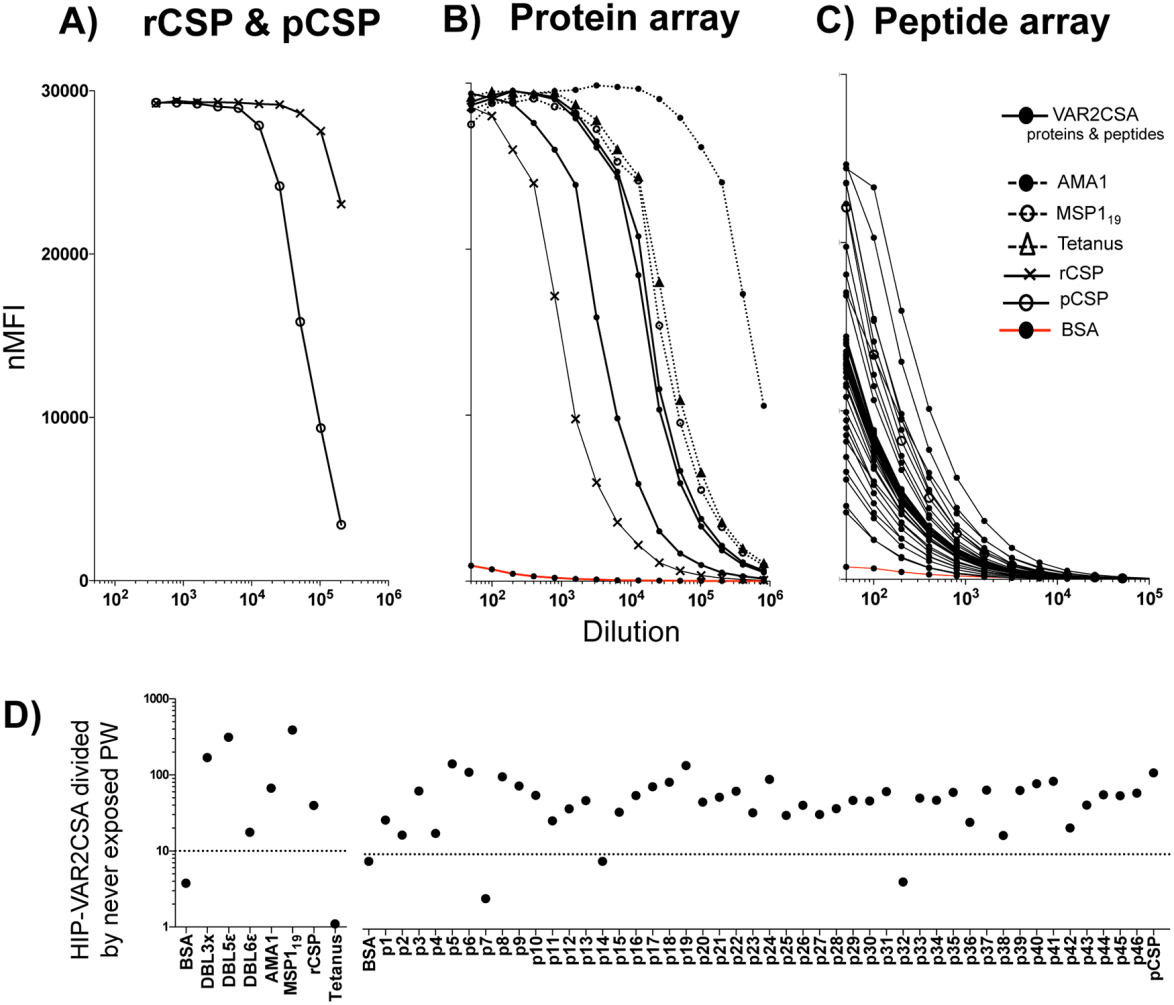
Antigens coupled on beads were highly recognized by IgGs in hyperimmune samples compared with negative controls. A) nMFIs obtained from 10 two-fold dilutions (1:400 to 1:20,000) of CSP hyperimmune serum measured against the CSP recombinant protein (rCSP) and peptide (pCSP). B) and C) nMFIs obtained from 15 two-fold dilutions (1:50 to 1:1.6x10^6^) of VAR2CSA hyperimmune plasma (HIP-VAR2CSA) measured against the protein array (3 VAR2CSA recombinant domains + AMA1, MSP1_19_, rCSP, tetanus toxin) and the peptide array (46 VAR2CSA-based peptides + pCSP). D) nMFIs measured in HIP-VAR2CSA divided by mean nMFIs measured in 5 pregnant Spanish women never exposed to malaria (PW) (dilution 1:100 for peptides array and 1:400 for proteins array).

Correct coupling of antigens on beads was confirmed by measuring the IgG recognition from hyperimmune samples. Beads coupled with rCSP and pCSP showed maximum nMFIs (≈30,000) when the least diluted pool from RTS,S/AS02-vaccinated children was used (10 points two-fold dilution curve, 1:400 to 1:20,000) (Fig 1A). The analysis of the protein array (3 VAR2CSA recombinant domains + AMA1, MSP1_19_, rCSP and tetanus toxin) and peptide array (46 VAR2CSA-based peptides + pCSP) against HIP-VAR2CSA resulted in a 15 points two-fold dilution curve (1:50 to 1:1.6x10^6^) for each antigen (Fig 1B and 1C) and nMFIs at least 10 times higher compared with the recognition by negative controls (except p7, p14 and p32 with a quotient <10) (Fig 1D). High nMFIs measured in hyperimmune pools compared with lower recognition by plasmas from negative controls and low background confirmed that all beads were properly coupled.

### Multiplexing does not affect IgG antibody reactivity

**Fig 2:**
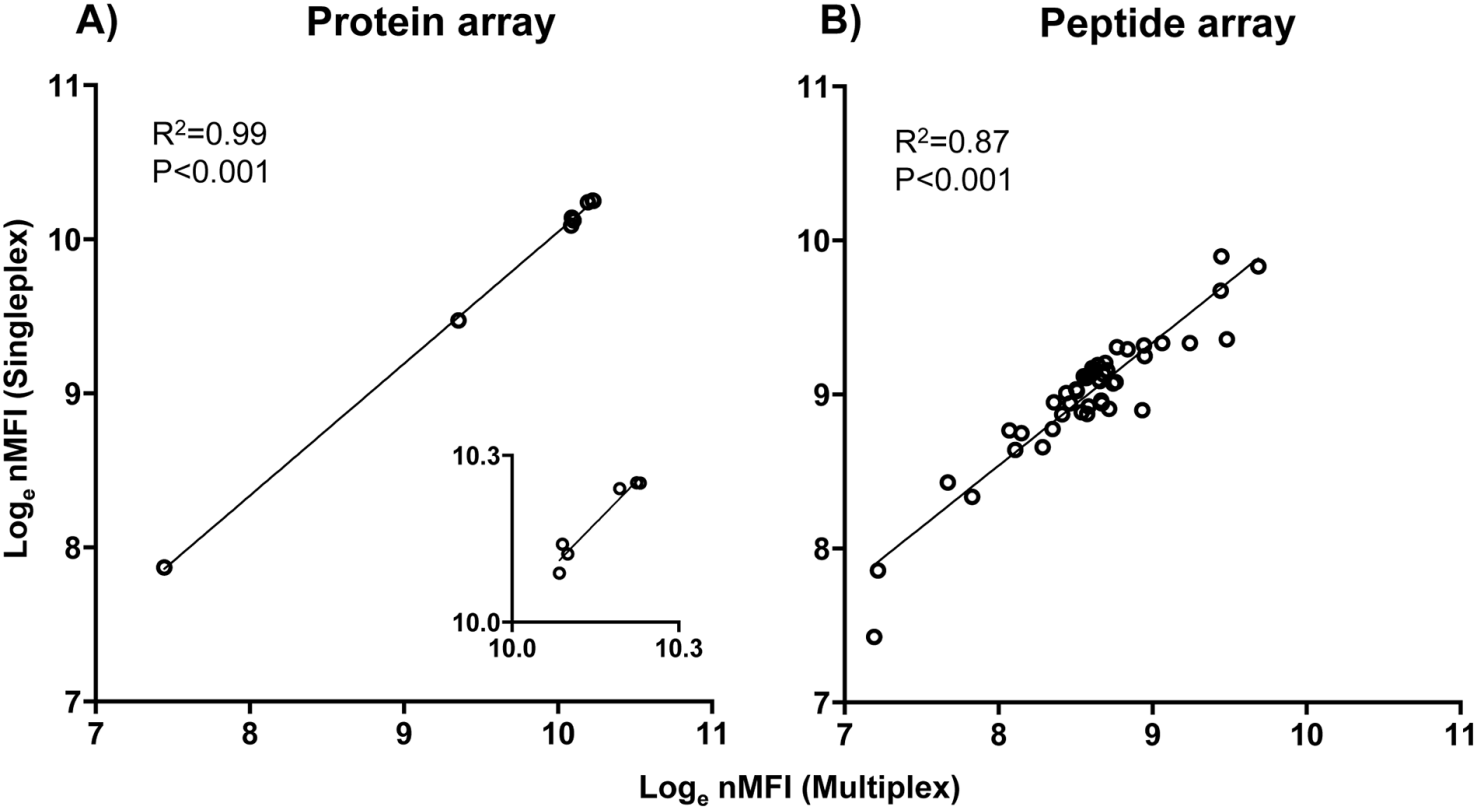
Correlation of nMFIs measured in VAR2CSA hyperimmune plasma in singleplex and multiplex. A) Protein array (3 VAR2CSA recombinant domains + AMA1, MSP1_19_, rCSP and tetanus toxin). B) Peptide array (46 VAR2CSA-based peptides + pCSP). Each dot corresponds to the nMFI value for each antigen of the array.

nMFIs measured in HIP-VAR2CSA using the singleplex beads were highly correlated with nMFI values obtained in multiplex for all antigens tested (protein array [3 VAR2CSA recombinant domains + AMA1, MSP1_19_, rCSP and tetanus toxin] and peptide array [46 VAR2CSA-based peptides + pCSP]) (Fig 2). Pearson′s rank correlation coefficient was R^2^ = 0.99 (p < 0.001) for the protein array and R^2^ = 0.87 (p < 0.001) for the peptide array. These results indicate that in the multiplex format the individual antigens on the beads do not compete for the available antibodies in the plasma sample.

### Intra and inter-assay variation

The intra-assay variation (mean CV of replicates from 7 plasma samples per plate) ranged from 2.6% to 6.5% for the protein array (3 VAR2CSA recombinant domains + AMA1, MSP1_19_, rCSP and tetanus toxin) and from 3.8% to 7.6% for the peptide array (46 VAR2CSA-based peptides + pCSP) in a total of 37 plates. The inter-assay variation (variability of positive pool [HIP-VAR2CSA] between 37 plates) was 3.8% for the protein array and 16.7% for the peptide array. The normalization strategy reduced the variability between the 37 plates as shown by a decrease of CV obtained for the median MFI of anti-tetanus IgG after normalization (CV reduction from 4.4% to 2.3%) (Figure B in S1 File).

### IgGs against VAR2CSA recombinant proteins and peptides in individuals exposed and non-exposed to malaria

**Fig 3:**
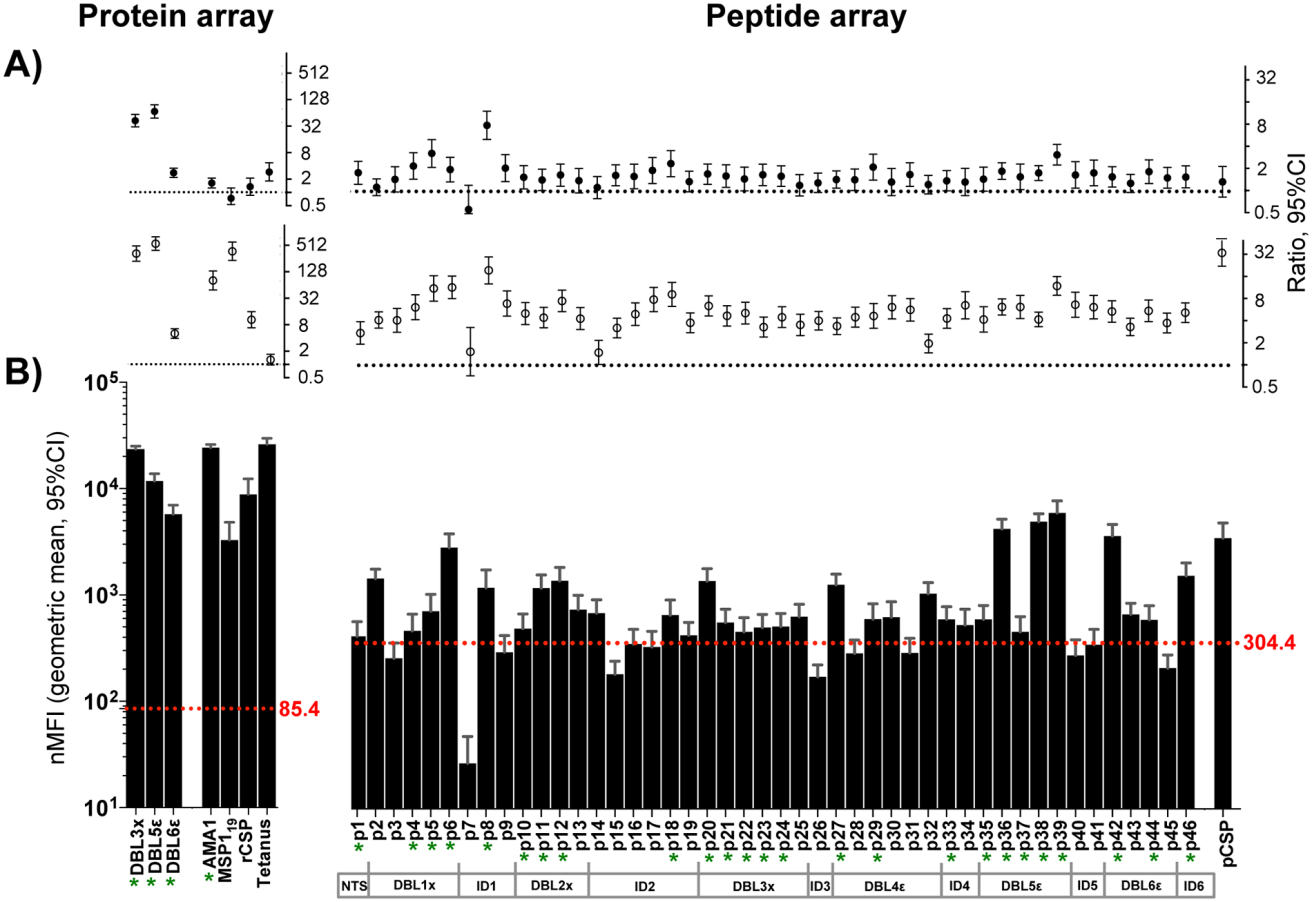
IgG antibodies measured in plasma from VAR2CSA-immune, pregnant Mozambican women compared with plasma from Spanish individuals and Mozambican men. **A)** Ratio of nMFIs against the protein array (3 VAR2CSA recombinant domains + AMA1, MSP1_19_, rCSP and tetanus toxin) and the peptide array (46 VAR2CSA-based peptides + pCSP) measured in VAR2CSA-immune pregnant Mozambican women and Spanish individuals (open circle) or Mozambican men (black circle), obtained by linear regression. T-bars correspond to the 95% confidence interval (CI). B) Bars represent the mean nMFIs from VAR2CSA-immune pregnant women. T-bars correspond to the 95% CI. Red dashed line represents the mean nMFI from BSA plus 3 standard deviations (SD) (BSA reactivity threshold). Asterisk indicates that IgG level from VAR2CSA-immune pregnant women was above the BSA reactivity threshold and statistically higher from both never exposed individuals and exposed men (p < 0.05 by linear regression).

IgGs from 106 pregnant Mozambican women immune against VAR2CSA, assessed by cytometry against native VAR2CSA expressed on CS2 parasite line, recognized all the recombinant proteins and 34/46 peptides at levels above BSA recognition (mean nMFI from each malaria antigen above mean nMFI from BSA plus 3 SD). All antigens were recognized at higher levels by plasma IgGs from VAR2CSA-immune pregnant women than from never exposed individuals (p < 0.05), with the exception of peptide 7 (p = 0.269) and tetanus toxin (p = 0.097). Levels of recognition of 34/46 of VAR2CSA peptides and all recombinant proteins were higher among VAR2CSA-immune, pregnant Mozambican women than among Mozambican men exposed to malaria (p < 0.05). The non-pregnancy-specific malaria antigens were equally recognized by IgGs from VAR2CSA-immune pregnant women and men exposed to malaria (rCSP, p = 0.206; pCSP, p = 0.217 and MSP1_19_, p = 0.247). Overall, 25 out of 46 VAR2CSA peptides (3 peptides from DBL1X, 3 from DBL2X, 5 from DBL3X, 2 from DBL4Ɛ, 5 from DBL5Ɛ, 2 from DBL6Ɛ and 5 from NTS and ID regions) and all VAR2CSA recombinant proteins (DBL3X, DBL5Ɛ and DBL6Ɛ) were recognized by IgGs from VAR2CSA-immune pregnant women above the BSA threshold and at higher levels than by plasma from never exposed Spanish individuals and exposed Mozambican men (Fig 3A and 3B).

### Dried blood spots as a source of IgG antibodies

**Fig 4:**
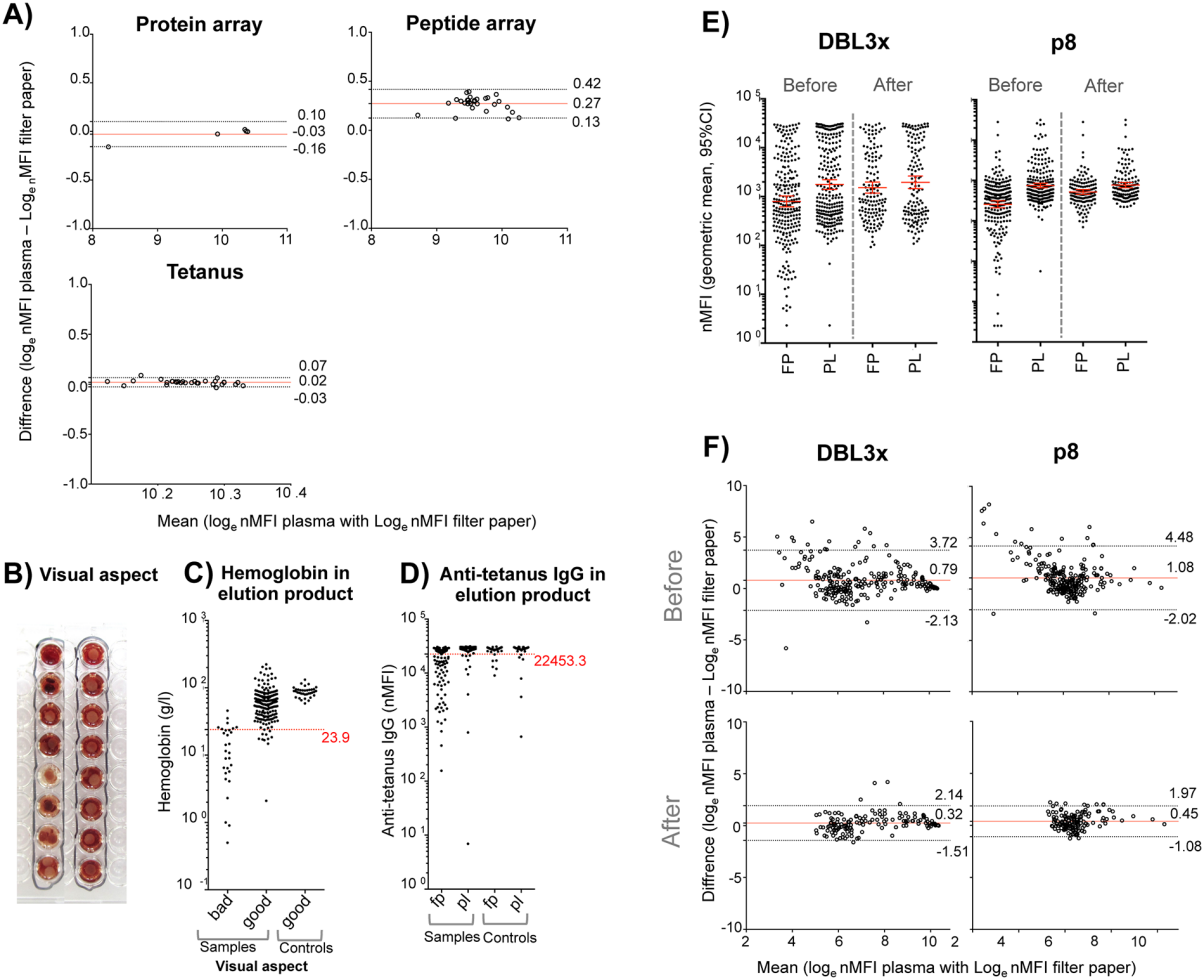
Performance of the multiplex assay to measure IgGs eluted from dried blood spots. A) Bland-Altman plots showing the agreement of nMFIs from VAR2CSA-hyperimmune pregnant women (HIP-VAR2CSA) measured in cryopreserved plasma (PL) and the corresponding elution product from dried blood spots (DBS) against protein array (3 VAR2CSA recombinant domains + AMA1, MSP1_19_, rCSP and tetanus toxin) and peptide array (subset comprised by 15 VAR2CSA-based peptides + pCSP) and from pregnant Spanish women against tetanus toxin. Red lines indicate the bias (mean of the differences) and dashed lines the 95% confidence level of agreement (CL). B) Visual aspect after DBS reconstitution. Reddish-brown spots against a pale background indicate inappropriate elution. C) Dot-plot representing the hemoglobin (g/dl) measured in the product of DBS elution from pregnant Mozambican and Spanish (control) women separated by quality of visual inspection. Red dashed line represents the upper quartile of hemoglobin level measured in the eluted DBS from samples with inappropriate visual aspect. D) Dot-plot representing nMFIs against the tetanus toxin measured in the product of DBS elution and corresponding cryopreserved plasma from pregnant Mozambican women and Spanish controls. Red dashed line represents the lower quartile of anti-tetanus IgG measured in the product of DBS elution among samples with appropriate visual aspect and hemoglobin levels. E) Dot plots of nMFIs against DBL3X and p8 from VAR2CSA inter-domain 1 measured in the product of DBS elution and in corresponding cryopreserved plasma from pregnant Mozambican women before and after discarding DBS of inappropriate quality. Red line represent nMFI means and T-bars the 95% confidence interval (CI) and, F) agreement represented by Bland-Altman plots.

The agreement between nMFIs against the protein array (3 VAR2CSA recombinant domains + AMA1, MSP1_19_, rCSP and tetanus toxin) and the peptide array (subset comprised by 15 VAR2CSA-based peptides + pCSP) obtained with cryopreserved plasma pooled from women with high levels of IgGs against VAR2CSA and the correspondent elution product of freshly prepared DBS showed a bias of -0.03 (CLw = 0.26) for protein array and 0.27 (CLw = 0.29) for peptide array (Fig 4A). Similar agreement was obtained for anti-tetanus toxin nMFIs measured in samples from 37 pregnant Spanish women (bias = 0.02 and CLw = 0.1) (Fig 4A). Twenty-nine out of the 240 DBS from pregnant Mozambican women had an inappropriate visual aspect after spot reconstitution (reddish-brown spots against a pale background, Fig 4B). Elutions from additional 11 DBS revealed low hemoglobin levels (below the highest quartile of samples with inappropriate visual aspect, Fig 4C), and a further 50 revealed low anti-tetanus toxin levels (below the lowest quartile, Fig 4D). Altogether, 63% (150/240) of DBS collected from pregnant Mozambican women yielded appropriate elution based on visual inspection of spot reconstitution as well as adequate hemoglobin levels and anti-tetanus toxin nMFIs measured in the eluted product. The range of Bland-Altman plot CLw obtained from protein array before exclusion of improperly eluted samples varied from 5.25 for AMA1 to 6.59 for MSP1_19_ and decreased after exclusion to 2.16 for AMA1 to 3.94 for DBL5Ɛ. Regarding the peptide array, the range before exclusion was from 4.57 for pCSP to 7.06 for p44 and also decreased after exclusion (2.75 for pCSP to 4.48 for p44) (Fig 4E and 4F and Table 2). All freshly prepared samples from 37 pregnant Spanish women (control) showed appropriate elution by visual inspection, hemoglobin levels above the threshold (Fig 4C), and the majority (84% [31/37]) showed nMFIs above the tetanus threshold (Fig 4D).

**Table 2:** Agreement of nMFIs measured in plasma and corresponding dried blood spots before and after discarding improperly eluted samples.

	BEFORE (n = 240)			AFTER (n = 150)		
	bias	CL	CLw	bias	CL	CLw
**Protein array**						
DBL3x	0.79	(-2.13; 3.72)	5.85	0.32	(-1.51; 2.14)	3.65
DBL5ε	0.64	(-2.44; 3.72)	6.15	0.15	(-1.82; 2.12)	3.94
DBL6ε	1.20	(-1.43; 3.82)	5.26	0.72	(-0.60; 2.05)	2.65
AMA1	0.57	(-2.06; 3.19)	5.25	0.29	(-0.79; 1.37)	2.16
MSP119	1.34	(-1.96; 4.63)	6.59	0.75	(-1.07; 2.57)	3.64
rCSP	1.53	(-1.69; 4.76)	6.45	0.89	(-0.64; 2.43)	3.07
**Peptide array**						
p1	0.84	(-1.86; 3.53)	5.39	0.35	(-1.41; 2.12)	3.53
p4	0.81	(-1.69; 3.31)	5.00	0.38	(-1.37; 2.13)	3.5
p5	0.36	(-2.62; 3.34)	5.96	-0.17	(-1.94; 1.60)	3.54
p6	1.30	(-1.82; 4.41)	6.22	0.72	(-0.99; 2.42)	3.41
p8	1.08	(-2.02; 4.18)	6.21	0.45	(-1.08; 1.97)	3.05
p10	-0.01	(-2.75; 2.73)	5.47	-0.59	(-2.22; 1.04)	3.26
p12	0.38	(-2.52; 3.28)	5.80	-0.23	(-1.70; 1.25)	2.95
p18	0.18	(-2.81; 3.17)	5.98	-0.39	(-2.25; 1.46)	3.71
p20	0.78	(-2.29; 3.86)	6.15	0.16	(-1.38; 1.70)	3.08
p22	-0.05	(-3.05; 2.06)	5.11	-1.05	(-2.42; 0.33)	2.75
p24	0.23	(-2.35; 2.82)	5.17	-0.24	(-1.72; 1.24)	2.96
p33	0.53	(-1.98; 3.03)	5.02	-0.01	(-1.40; 1.42)	2.82
p36	0.21	(-3.08; 3.49)	6.57	-0.37	(-2.28; 1.54)	3.82
p37	0.30	(-2.45; 3.06)	5.51	-0.22	(-2.16; 1.71)	3.87
p44	0.58	(-2.95; 4.11)	7.06	-0.06	(-2.30; 2.18)	4.48
pCSP	0.29	(-1.99; 2.57)	4.57	-0.18	(-1.53; 1.18)	2.71

### Definition of seropositivity

**Fig 5:**
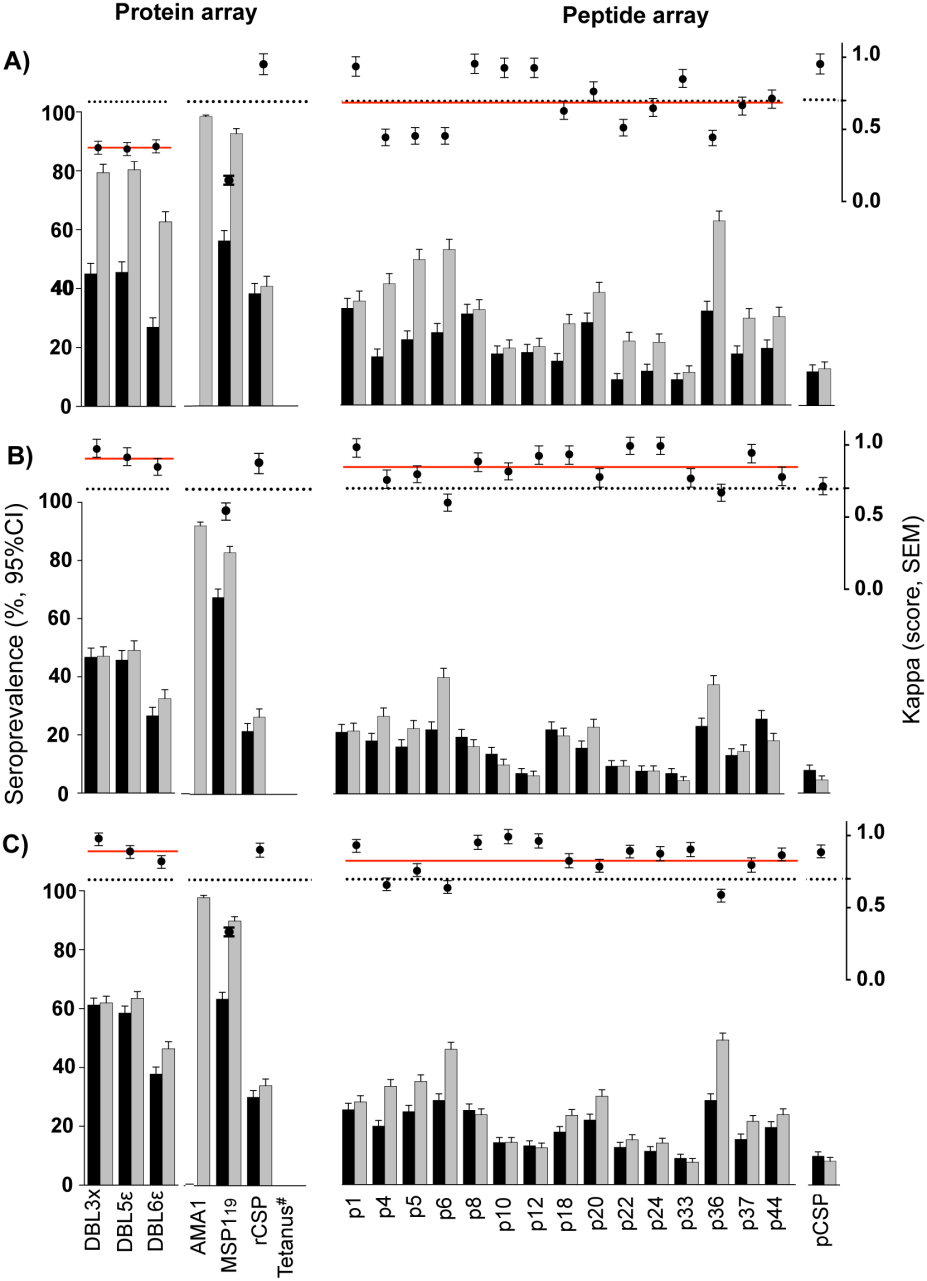
Agreement of seroprevalences defined by finite mixture models and pregnant women never exposed to malaria in periods of different malaria transmission intensity. Three epidemiological scenarios were considered to test the agreement of seroprevalences against the protein array (3 VAR2CSA recombinant domains + AMA1, MSP1_19_, rCSP and tetanus toxin) and the peptide array (subset comprised by 15 VAR2CSA-based peptides + pCSP): A) high malaria intensity; B) low malaria intensity; C) both periods together (high & low). Black bars correspond to seroprevalence obtained by finite mixture models (FMM), grey bars to seroprevalence obtained by pregnant women never exposed to malaria and T-bars indicate the 95% confidence interval (CI). Dots are the Kappa statistic of agreement of seroprevalences obtained by both methods for each antigen, red line represents the mean of Kappa score, dashed line represents 0.7 Kappa score threshold for good agreement and T-bars represent standard errors of the mean (SEM).

Seroprevalence against VAR2CSA antigens (DBL3X, DBL5Ɛ, DBL6Ɛ and 15 VAR2CSA-based peptides) obtained by cutoffs derived from never exposed pregnant Spanish women and FMM (Table C in S1 File) agreed fairly for protein array (mean Kappa score [standard error of the mean—SEM] = 0.35 [0.05], p < 0.001) and substantially for peptide array (mean Kappa score [SEM] = 0.69 [0.07], p < 0.001) in plasma from 204 pregnant Mozambican women from a period of high malaria intensity, 2003–2005 (Fig 5A, Table D in S1 File). In contrast, the two arrays agreed almost perfectly in plasma from 240 pregnant Mozambican women collected during the period of low malaria intensity between 2010 and 2012 (mean Kappa score [SEM] of protein array = 0.93 [0.06], p < 0.001 and of peptide array = 0.85 [0.06], p < 0.001 (Fig 5B, Table D in S1 File). When samples from both periods (high & low) were pooled, the arrays also agreed almost perfectly (mean Kappa score [SEM] of protein array = 0.90 [0.05], p < 0.001 and peptide array = 0.83 [0.05], p < 0.001) (Fig 5C, Table D in S1 File). Regarding the non-pregnancy specific malaria antigens, good and almost perfect agreement of seroprevalences was observed for the less immunogenic pCSP (high: Kappa [SEM] = 0.95 [0.07]; low: 0.72 [0.06]; high & low: 0.89 [0.05]; p < 0.001) and rCSP (high: 0.95 [0.07]; low: 0.87 [0.06]; high & low: 0.91 [0.05]; p < 0.001). In contrast, the more immunogenic AMA1 (Kappa = 0 in all scenarios) and MSP1_19_ (high: 0.16 [0.04]; low: 0.56 [0.06]; high & low: 0.34 [0.04]; p < 0.001) failed to produce a good agreement between arrays. It was not possible to define seropositivity against tetanus toxin because all individuals had high IgG levels and no population could be identified as negative.

### Anti-VAR2CSA IgGs measured in pregnant Mozambican women from two periods of different malaria transmission intensity

**Fig 6:**
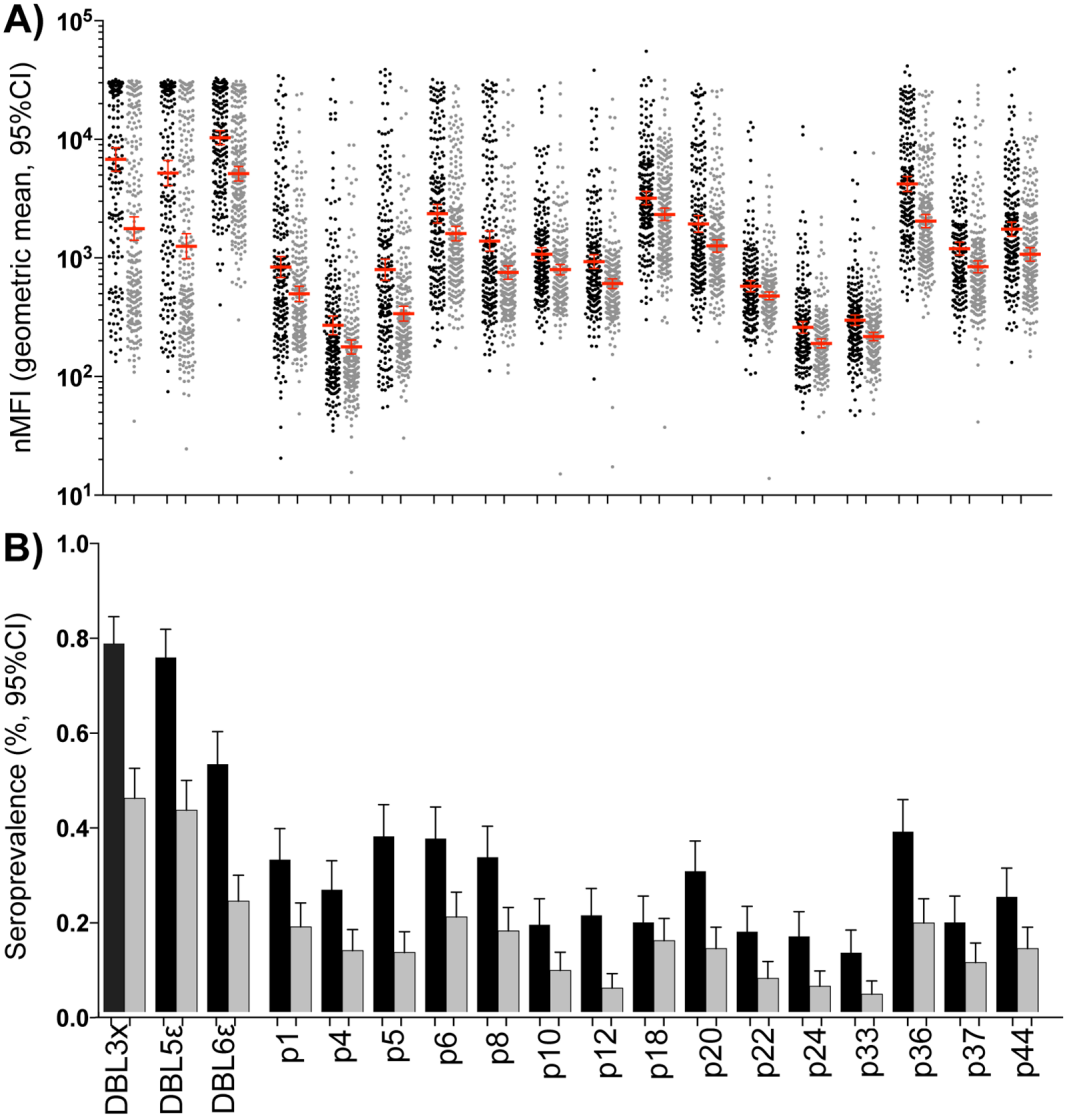
IgGs in pregnant women from two periods of different malaria transmission intensity. A) The dot plot shows the nMFIs against the protein array (DBL3X, DBL5Ɛ, and DBL6Ɛ) and the peptide array (subset comprised by 15 VAR2CSA-based peptides) in periods of high (2003–2005; black dots) and low (2010–2012; grey dotes) malaria transmission intensity. Dots represent nMFI of each pregnant woman, red lines correspond to geometric mean and T-bars represent the 95% confidence interval (CI). B) Seroprevalence obtained in periods of high and low malaria intensity (cutoff: FMM high & low). Black bars correspond to seroprevalence in the period of high intensity, grey bars to seroprevalence in the period of low intensity and T-bars to 95%CI. Differences of nMFIs or seroprevalences between high and low transmission periods assessed by linear and logistic regressions were statistically significant (p < 0.05) for all antigens, except for seroprevalences against p18 (p = 0.265).

Prevalence of malaria infection was assessed by qPCR in peripheral and placental blood from pregnant Mozambican women (Table 1). In samples from 2003 to 2005, prevalence was 39% (79/204). In samples from 2010 to 2012, prevalence was 5% (12/240). nMFIs against the protein array (DBL3X, DBL5Ɛ, and DBL6Ɛ) and the peptide array (subset comprised by 15 VAR2CSA-based peptides) were higher in women from 2003–2005 (high malaria intensity) than from 2010–2012 (low malaria intensity) (Fig 6A). The ratio of mean nMFI measured in women during high malaria intensity and mean nMFIs measured in women during low malaria intensity assessed by linear regression ranged from 2.05 (95%CI: 1.68, 2.49 for DBL6Ɛ) to 4.57 (95%CI: 3.28, 6.37 for DBL5Ɛ) for proteins and from 1.21 (95%CI: 1.06, 1.38 for p22) to 2.34 (95%CI: 1.82, 2.99 for p5) for peptides. The same difference between malaria intensity periods was observed for seroprevalences when we used the FMM as seropositivity cutoff in all samples (high & low) (Fig 6B). The odds ratio of seropositivity between high malaria intensity and low intensity assessed by logistic regression ranged from 3.64 (95%CI: 2.41, 5.51 for DBL6Ɛ) to 5.91 (95%CI: 3.68, 9.47 for DBL3X) for protein array and from 1.33 (95%CI: 0.81, 2.19 for p18) to 4.45 (95%CI: 2.36, 8.36 for p12) for peptide array. Differences in nMFIs and seroprevalences were statistically significant (p < 0.05) between study periods for all antigens, except for seroprevalences against p18 (p = 0.265).

## Discussion

Here we developed a bead-based immunoassay that multiplexes the measurement of IgGs against synthetic peptides from the *P*. *falciparum* pregnancy-specific antigen VAR2CSA. The assay allows the detection of antibody responses against 46 new VAR2CSA peptides and additionally 3 recombinant domains (DBL3X, DBL5Ɛ and DBL6Ɛ). All VAR2CSA recombinant domains and 25 peptides coated onto beads were more highly recognized by IgGs from plasma of malaria-exposed pregnant women than from Spanish individuals and malaria-exposed Mozambican men, in accordance with the pregnancy-restricted exposure to VAR2CSA-expressing malaria parasites. Similar nMFI values were obtained when coated beads were tested alone or in combination, thus discarding interference by multiplexing the measurements. Intra-assay variation was 4.3% and inter-assay variation was 3.8% for protein array, whereas for peptide array it was 4.9% and 16.7%, which are acceptable ranges [47]. Moreover, plate-to-plate variation decreased after normalization with the positive control.

Results of this study confirm that DBS can be an alternative to plasma for studies on malaria serology during pregnancy [32,45]. However, it is necessary to discard samples in inappropriate conditions due to incorrect handling or storage of blood samples on filter papers. Although this can be achieved by visual inspection of the elution product after spot reconstitution [32], subjectivity can be avoided by quantitative approaches based on the measurement of hemoglobin levels and anti-tetanus IgGs in the elution product.

This study also showed that a two-component finite mixture model of seronegatives and seropositives can be fitted to the population from malaria endemic areas to assess the serological status of pregnant women, as has been done for seroprevalences to general malaria antigens (usually AMA1 and MSP1_19_) from populations composed of all age groups [50]. Such an approach can be applied in scenarios of heterogeneous antibody distributions where a subgroup of the study population is seronegative and another seropositive avoiding additional sampling of malaria never exposed pregnant women, which could be logistically complex. Importantly, seropositivity cutoffs to general malaria antigens (e.g. AMA1 and MSP1_19_) must be obtained from means of never exposed pregnant women as a consequence of the absence of younger age groups. In scenarios of extreme transmission (very low or very high), discrimination of both populations by FMM may be inaccurate due to an overlap or absence of seropositive and seronegative groups [51] and cutoffs defined based on means of never exposed pregnant women should be considered. Together with malaria transmission intensity, characteristics of the study population (percentage of primigravidae) or antigens (immunogenicity and longevity of antibody responses) can have an important influence on the performance of FMM and must be taken into account for a correct interpretation of the results.

Among the antigens used in this study, 3 serological profiles were observed. Merozoite (AMA1, MSP1_19_) and circumsporozoite (CSP recombinant and peptide) antigens were not recognized by Spanish individuals but similarly highly recognized by malaria-exposed men and pregnant women. Tetanus toxin was highly recognized by malaria exposed and non-exposed pregnant women as a consequence of vaccination required during pregnancy [52]. VAR2CSA domains and 25 out of 46 peptides were recognized by VAR2CSA-immune pregnant women but not by individuals never exposed to malaria and exposed men, in line with the specificity of VAR2CSA expressed by placental parasites [10,53]. Different dilution curves obtained against VAR2CSA-derived antigens suggested that both arrays were composed of antigens with different degrees of immunoreactivity. DBL3X and DBL5Ɛ domains were more recognized by malaria-exposed pregnant women than DBL6Ɛ, in accordance with previous studies showing that DBL3X and DBL5Ɛ are more conserved [24,54] than the C-terminal domain DBL6Ɛ [55,56]. Aminoacid variability obtained by the alignment of VAR2CSA sequences from parasites in different geographic area also showed low variability for DBL3X and DBL5Ɛ (entropy_DBL3X_ = 0.197, entropy_DBL5Ɛ_ = 0.208) compared with DBL6Ɛ (entropy = 0.522). Interestingly, among the 25 peptides selected as highly immunoreactive and pregnancy-specific, 10 (40%) were from DBL3X and DBL5Ɛ domains, also supporting the existence of conserved epitopes in these domains [24,54].

The VAR2CSA multiplex assay developed in this study was used to measure antibodies from a set of samples from pregnant Mozambican women collected between 2003 and 2012. This period was characterized by a substantial decline of malaria prevalence paralleled by a reduction of antimalarial antibody levels [21]. Here, by applying the FMM to this IgG heterogeneous population of pregnant women we were able to identify a high percentage of seropositive individuals in the period of high transmission (2003–2005) compared with a low percentage of seropositives in the period of low transmission (2010–2012), demonstrating the ability of this immunoassay to distinguish different malaria transmission intensity periods.

In conclusion, the VAR2CSA multiplex technology allows the measurement of pregnancy specific antibodies against a set of 25 VAR2CSA new peptides and 3 recombinant proteins (DBL3X, DBL5Ɛ, and DBL6Ɛ) in small volumes of plasma or DBS using a high-throughput approach. Anti-VAR2CSA IgG levels and seroprevalences assessed by FMM cutoff were related with the intensity of malaria in pregnancy, suggesting this technology as a valuable tool for investigating correlates of protection and identifying serological markers of exposure.

## Supporting information

S1 File **Figure A**. **Entropy plot of the multi-sequence alignment and peptide position**. Bars correspond to Shannon entropy values calculated on the multiple sequence alignment of 18 VAR2CSA aminoacid sequences from field isolates. VAR2CSA domains and regions (NTS: N-terminal segment; DBL, Duffy-binding like; ID: inter-domain region) are indicated. Dotted horizontal lines indicate second tercile of all Shannon entropy values (0.43). Green-lines indicate each peptide position and correspondent Shannon entropy mean (standard deviation [SD]). **Figure B**. **Effect of normalization on plate-to-plate variation**. Coefficient of variation (CV) before (A) and after (B) normalization by positive pool, as assessed by the median MFI of anti-tetanus toxin IgG measured in plasma from 7 pregnant women from Mozambique per plate in 37 consecutive plates. Black dots correspond to MFI of each women and red line to median MFI from 7 women per plate. **Table A. Geographic origin, Genebank accession number and reference of 18 VAR2CSA sequences used in the alignment. Table B. Amino acid sequences of 46 VAR2CSA peptides (N-terminal to C-terminal). Table C. Seropositivity thresholds obtained by pregnant Spanish women never exposed to malaria and finite mixture models in high, low and high & low malaria transmission intensity periods. Table D. Seroprevalences defined by pregnant Spanish women never exposed to malaria and finite mixture models in pregnant women from high, low and both (high & low) malaria transmission periods and correspondent kappa agreement. Text A. Peptides design. Text B. Coupling of microspheres. Text C. Luminex assay to measure total IgG**.
(DOCX)
