# Supplementary material for: Multiplexing detection of IgG against Plasmodium falciparum pregnancy-specific antigens
Source: PLoS One. 2017 Jul 17;12(7):e0181150. doi: 10.1371/journal.pone.0181150 (PMC5513451; doi:10.1371/journal.pone.0181150)
Supplement: S1 File — Figure A. Entropy plot of the multi-sequence alignment and peptide position. Bars correspond to Shannon entropy values calculated on the multiple sequence alignment of 18 VAR2CSA aminoacid sequences from field isolates. VAR2CSA domains and regions (NTS: N-terminal segment; DBL, Duffy-binding like; ID: inter-domain region) are indicated. Dotted horizontal lines indicate second tercile of all Shannon entropy values (0.43). Green-lines indicate each peptide position and correspondent Shannon entropy mean (standard deviation [SD]). Figure B. Effect of normalization on plate-to-plate variation. Coefficient of variation (CV) before (A) and after (B) normalization by positive pool, as assessed by the median MFI of anti-tetanus toxin IgG measured in plasma from 7 pregnant women from Mozambique per plate in 37 consecutive plates. Black dots correspond to MFI of each women and red line to median MFI from 7 women per plate. Table A. Geographic origin, Genebank accession number and reference of 18 VAR2CSA sequences used in the alignment. Table B. Amino acid sequences of 46 VAR2CSA peptides (N-terminal to C-terminal). Table C. Seropositivity thresholds obtained by pregnant Spanish women never exposed to malaria and finite mixture models in high, low and high & low malaria transmission intensity periods. Table D. Seroprevalences defined by pregnant Spanish women never exposed to malaria and finite mixture models in pregnant women from high, low and both (high & low) malaria transmission periods and correspondent kappa agreement. Text A. Peptides design. Text B. Coupling of microspheres. Text C. Luminex assay to measure total IgG. (DOCX) [file pone.0181150.s001.docx]

**Multiplexing detection of IgG against *Plasmodium falciparum* pregnancy-specific antigens**

**Supporting information**

**Text A. Peptides design**

Peptides were designed after alignment by Clustal W of 18 VAR2CSA full-length sequences from *Plasmodium falciparum* isolates of different geographic origins (Asia, Africa, Central and South America) (S1 Table). Shannon entropy values (measure of aminoacid diversity in each position of the alignment) [1] were obtained in Bioedit and a threshold of 0.43 (second tercile) was defined to distinguish the highly polymorphic regions from the conserved and semi-conserved regions (S1 Fig). Briefly, peptides correspond to sequences of at least 20 sequential aminoacids with an entropy value bellow the second tercile. A maximum of 2 positions with entropy values above threshold interspersed between conserved regions were included. In those cases with a position having more than 2 polymorphisms, the most frequent aminoacid was selected (random selection in case of equal prevalence). In those peptides less than 35 aminoacids-long (recommended length for optimal coupling [2,3]) polymorphic lateral positions were also included.

**Text B. Coupling of microspheres**

Two hundred μl of beads (2,5x10^6^) were transferred into a 1.5 mL eppendorf tube and resuspended by sonication and vortexing. The supernatant was removed after precipitation of the beads by a magnetic separator during 60 seconds. Beads were washed twice with 250 μl of distilled water and pellets were resuspended in 80 μl of activation buffer (0.1 M NaH2PO4, pH 6.2). Sulfo-NHS (N-hydroxysulfosuccinimide) and EDC (1-Ethyl- 3-[3-dimethylaminopropyl]carbodiimide hydrochloride) (Pierce, Thermo Fisher Scientific Inc., Rockford, IL) were simultaneously added to reaction tubes at 5 mg/mL each in activation buffer, and reaction tubes were incubated at room temperature with gentle agitation, protected from light for 20 minutes. Activated beads were washed twice with 250 μl of coupling buffer (MES 50 mM, 2-[N-morpholino] ethanesulfonic acid monohydrate pH 5, Sigma- Aldrich). Proteins and peptides were added to correspondent beads and volume was adjusted with coupling buffer until a maximum of 400 μl. Beads and antigens were vortexed, sonicated and then incubated over night at 4ºC in the dark, with shaking. Coupled beads were blocked with 500 μl of 1% bovine serum albumin (BSA) in PBS for 30 minutes on a shaker at room temperature, avoiding light and then washed twice with 500 μl of assay buffer (1% BSA, 0.05% sodium azide in PBS filtrated) and resuspended in 400 μl of the same buffer. Beads were quantified on a Guava PCA desktop cytometer (Guava, Hayward, CA), and stored at 4°C in the dark.

**Text C. Luminex assay to measure total IgG**

Immediately before use, stock suspensions of antigen-coated microspheres were thoroughly resuspended by vortexing and sonication. Frozen plasma samples were thawed at room temperature, mixed by vortexing, and spin at 16000 g for 5 minutes to remove particles. 50 μl of diluted microspheres (1000 microspheres/analyte/well) were added to a 96-well mylar flat-bottom plate following the addition of 50 μl of diluted plasma in duplicates to a final concentration of 1:400 for protein array and 1:100 for peptide array and incubated for 1 hour in agitation, protected from light at room temperature. After incubation, the plates were washed 3 times with 200 μl of washing buffer (0.05% Tween 20 in PBS) by pelleting in a magnetic 96 well separator. 100 μl of biotinylated anti-human IgG (Sigma, Tres Cantos, Spain) diluted 1:1000 in assay buffer was added to each well, and plates were incubated for 45 minutes in agitation, protected from light at room temperature. After the incubation period, the plates were washed as before and 100 μl of streptavidin-conjugated R-phycoeryhtrin (Invitrogen, Carlsbad, CA) at a 1:1000 dilution in assay buffer was added and incubated for 25 minutes in the same conditions previously mentioned. Finally the plates were washed as before and the beads were resuspended in 100 μl of assay buffer and analyzed using the Luminex® 100/200™ System.

**Figure A**. **Entropy plot of the multi-sequence alignment and peptide position**. Bars correspond to Shannon entropy values calculated on the multiple sequence alignment of 18 VAR2CSA aminoacid sequences from field isolates. VAR2CSA domains and regions (NTS: N-terminal segment; DBL, Duffy-binding like; ID: inter-domain region) are indicated. Dotted horizontal lines indicate second tercile of all Shannon entropy values (0.43). Green-lines indicate each peptide position and correspondent Shannon entropy mean (standard deviation [SD]).

**Figure B**. **Effect of normalization on plate-to-plate variation.** Coefficient of variation (CV) before (A) and after (B) normalization by positive pool, as assessed by the median MFI of anti-tetanus toxin IgG measured in plasma from 7 pregnant women from Mozambique per plate in 37 consecutive plates. Black dots correspond to MFI of each women and red line to median MFI from 7 women per plate.

**Table A.** **Geographic origin, Genebank accession number and reference of 18 VAR2CSA sequences used in the alignment.**

| **Geographic origin** | **Accession number** (protein) | **Reference** |
| --- | --- | --- |
| SE Asia | AAQ73930 | Kraemer and Smith, 2003 |
| SE Asia | AAQ73926 |  |
| SE Asia | AAQ73924 |  |
| SE Asia | ABS79813 | Bockhorst, et al, 2007 |
| SE Asia | ABS79814 |  |
| SE Asia | ABS79815 |  |
| SE Asia | ABS79816 |  |
| SE Asia | ABS79817 |  |
| E Africa | XP_001350415 | Gardner, et al, 2002 |
| E Africa | ABS79818 | Bockhorst, et al, 2007 |
| E Africa | ABS79819 |  |
| E Africa | ABS79820 |  |
| W Africa | ABS79821 |  |
| S America | ABS79822 |  |
| C America | HB3sc1-295 | http://www.broad.mit.edu |
| C America | HB3sc1-262 |  |
| C America | HB3var2csaA | http://www.cbs.dtu.dk/cgi-bin/webface |
| C America | HB3var2csaB |  |

**Table B.** **Amino acid sequences of 46 VAR2CSA peptides (N-terminal to C-terminal)**

**Table C. Seropositivity thresholds obtained by pregnant Spanish women never exposed to malaria and finite mixture models in high, low and high & low malaria transmission intensity periods**.

|  | **Never exposed PW*** |  | **FMM** | | |
| --- | --- | --- | --- | --- | --- |
|  |  |  | **high** | **low** | **High & low** |
|  | mean+3SD |  | mean+3SD | mean+3SD | mean+3SD |
| **Protein array** | |  |  |  |  |
| DBL3x | 1368.67 |  | 20202.95 | 1400.37 | 1447.82 |
| DBL5ε | 737.25 |  | 12602.27 | 969.02 | 1144.02 |
| DBL6ε | 8093.25 |  | 25800.74 | 9909.14 | 11220.63 |
| AMA1 | 5056.25 |  | 44542.36 | 48089.15 | 47910.02 |
| MSP1_19_ | 366.93 |  | 8670.87 | 1392.78 | 3869.55 |
| rCSP | 6259.87 |  | 7099.31 | 8104.68 | 7717.40 |
| Tetanus | 48703.14 |  | 40693.42 | 46539.19 | 43531.98 |
| **Peptide array** | |  |  |  |  |
| p1 | 1024.2 |  | 1298.71 | 1078.42 | 1237.06 |
| p4 | 226.38 |  | 730.88 | 335.73 | 448.75 |
| p5 | 603.13 |  | 2085.36 | 800.75 | 1003.13 |
| p6 | 1829.77 |  | 5667.09 | 3272.26 | 3516.23 |
| p8 | 1721.95 |  | 1951.08 | 1332.21 | 1609.94 |
| p10 | 1953.74 |  | 2288.22 | 1623.94 | 1938.41 |
| p12 | 1671.68 |  | 1924.04 | 1376.54 | 1557.93 |
| p18 | 5147.87 |  | 7941.2 | 4987.13 | 6426.76 |
| p20 | 2125.35 |  | 3235.13 | 2692.81 | 2882.63 |
| p22 | 914.71 |  | 1460.81 | 890.62 | 1052.43 |
| p24 | 418.46 |  | 613.22 | 406.68 | 502.15 |
| p33 | 620.18 |  | 717.24 | 478.38 | 577.00 |
| p36 | 2531.42 |  | 6866.72 | 3968.99 | 4966.87 |
| p37 | 1652.05 |  | 2604.36 | 1793.35 | 2186.54 |
| p44 | 2383.95 |  | 3797.17 | 1864.48 | 3063.01 |
| pCSP | 1008.19 |  | 1106.09 | 763.92 | 909.98 |
| PW, Pregnant women; FMM, Finite mixture model; SD, Standard deviation  *2 or less plasma samples were considered outliers and excluded by showing nMFI above decile 9 plus 1.5 times the inter-decile range for each antigen | | | | | |
|  |  |  |  |  |  |

**Table D.** **Seroprevalences defined by pregnant Spanish women never exposed to malaria and finite mixture models in pregnant women from high, low and both (high & low) malaria transmission periods and correspondent Kappa agreement.**

**References**

1. Valdar WS (2002) Scoring residue conservation. Proteins 48: 227-241.

2. Ambrosino E, Dumoulin C, Orlandi-Pradines E, Remoue F, Toure-Balde A, et al. (2010) A multiplex assay for the simultaneous detection of antibodies against 15 Plasmodium falciparum and Anopheles gambiae saliva antigens. Malar J 9: 317.

3. Fouda GG, Leke RF, Long C, Druilhe P, Zhou A, et al. (2006) Multiplex assay for simultaneous measurement of antibodies to multiple Plasmodium falciparum antigens. Clin Vaccine Immunol 13: 1307-1313.
